# Left atrial phasic function remodeling during its enlargement: a two-dimensional speckle-tracking echocardiography study

**DOI:** 10.1186/s12872-022-02672-z

**Published:** 2022-05-19

**Authors:** Chuyun Chen, Ying Yang, Wei Ma, Litong Qi, Baowei Zhang, Yan Zhang

**Affiliations:** 1 Department of Cardiology, Peking University First Hospital, 8 Xishiku St, Xicheng District, Beijing, 100034 China; 2 Echocardiography Core Lab, Institute of Cardiovascular Disease, Peking University First Hospital, 8 Xishiku St, Xicheng District, Beijing, 100034 China

**Keywords:** Atrial function, Speckle tracking, Hypertension, Phasic function, Left atrial remodeling

## Abstract

**Background:**

Left atrial (LA) size is often used as a surrogate marker of LA function in clinical practice, with larger atrial thought to represent a “dysfunctioning” atrium, since there is no accepted ‘gold’ standard to evaluate LA function. The exact relationship between LA size and phasic function, and whether LA dysfunction occur before LA enlargement (LAE) may be of clinical interest while have not been fully studied. Two-dimensional speckle-tracking echocardiography (2D STE) was showed a promising method in measuring LA physical deformation.

**Materials and methods:**

A community cohort of 715 subjects at cardiovascular disease high risk accepted comprehensive echocardiography. LA longitudinal phasic strain Sa (absolute peak strain during atrial contraction), Se (peak strain at early diastole) and Stot (total atrial strain = Sa + Se), representing contractile, conduit, and reservoir function respectively, were measured using off-line 2D STE software in apical 4 chamber view, and data were compared among groups at different LA size and between subgroups in normal LA size with and without hypertension (HT).

**Results:**

With LAE (from normal size, mild, moderate to severe LAE), the Stot (21.74 ± 5.97, 20.75 ± 4.99, 20.49 ± 5.27, 17.75 ± 4.71, respectively, ANOVA *P* = 0.003) and Sa (11.84 ± 3.92, 11.00 ± 3.29, 10.11 ± 2.57, 8.55 ± 2.88, respectively, ANOVA *P* < 0.001) reduced while Se had no change. Stot of Severe LAE group was significantly lower than that of Normal LA size group (*P* = 0.002). Sa of the three LAE groups were all significantly lower than that of Normal LA size group (*P* = 0.024, *P* = 0.002, *P* < 0.001, respectively). In normal sized LA subgroups, Stot (21.35 ± 5.91 vs. 23.01 ± 6.02, *P* = 0.008) and Se (9.51 ± 4.41 vs. 11.17 ± 4.89, *P* < 0.001) reduced in subjects with HT comparing with those without.

**Conclusion:**

LA phasic function remodeling occurs before LAE and continues with LAE, with reservoir, conduit and contractile function being affected unparalleled.

**Supplementary Information:**

The online version contains supplementary material available at 10.1186/s12872-022-02672-z.

## Background

The left atrium (LA) plays an integral role in cardiac performance by modulating left ventricular (LV) filling with its reservoir, conduit, and contractile functions [1]. In clinical practice, LA size, an essential component of echocardiographic parameters, is easily available and widely used as a surrogate marker of its function and regarded as a powerful predictor for adverse clinical outcomes of cardiovascular diseases (CVD) [2–8]. However, the application of a simple geometric model to a nonsymmetrical chamber in echocardiography to assess LA enlargement (LAE) is actually indirect and limited.

Recently, more and more attention is paid to LA function. Abundant evidences seemed to indicate that LA functional parameters might be surrogate markers of unfavorable cardiovascular outcomes as well [9–13]. Atrial strain, as an adjunctive measurement of LA function, is an emerging parameter of interest and has been shown to be less load dependent than traditional parameters [14–16]. However, much less data is available regarding direct relationship between LA size and its phasic function for there is currently no recognized ‘gold’ standard method in LA function assessment [15].

Therefore, utilizing two-dimensional speckle-tracking echocardiography (2D STE) our study aimed to assess LA phasic strain in subjects stratified as high-risk for CVD risk to explore the relationship between LA size and phasic function. We also aimed to elucidate any changes in LA phasic components during in LA remodeling spectrum and if normal LA structure dictates normal phasic function.

## Materials and methods

### Study population

A community cohort [17] screened for CVD and risk factors in urban Beijing was set up in 2005 and 1058 subjects were included. In 2009, participants were followed up through telephone invitation. Of the 1058 subjects, 154 were lost (14.6%), 60 died and 74 accepted telephone follow-up while refused on site examination, which made 770 subjects were screened on site. Detailed medical history of known CVD, cardiovascular risk factors, and medication history were recorded. Blood pressure was recorded in hypertension (HT) participants. Coronary heart disease (CHD) was defined as "hospitalization for myocardial infarction or/and history of percutaneous coronary intervention or coronary artery bypass graft". Height, weight, heart rate were recorded, and routine blood tests were performed. All 770 were at high risk of CVD defined as either one had cardiovascular events including myocardial infarction, stroke and peripheral arterial disease, or had at least 2 risk factors including age ≥ 50 years, smoking (current smoker), obesity (BMI ≥ 28, according to the definition of Chinese population [18]), HT, diabetes mellitus (DM) and hyperlipidemia (total cholesterol > 5.20 mmol/L).

All 770 subjects accepted comprehensive echocardiography examination, and the 2009 cross-sectional data of 770 subjects were used in this study. All participants provided written informed consent, and the study protocol was approved by the ethics committee of Peking University First Hospital. The study was conducted in accordance with the Declaration of Helsinki.

### Echocardiography exam

Comprehensive transthoracic echocardiography was performed on a Vivid 7 ultrasound machine (Vingmed-General Electric, Horten, Norway), using a 1.5–4 MHz phased array transducer. Echocardiographs (with 2D image frame rate over 50 frames/second) with 3 consecutive beats were digitally recorded and analyzed using a customized off-line analyzing software package (Echo PAC, version 110, Vingmed-General Electric). All images and measurements were acquired from the standard views according to the guidelines of the American Society of Echocardiography [19–21].

LA volume was measured using the biplane method of discs at end systole (LAVmax) and end diastole (LAVmin). LA volume index (LAVI) was calculated by dividing LAVmax to the body surface area (BSA) [22]. LAEF was defined as: [(LAVmax − LAVmin)/LAVmax] × 100. Pulmonary artery systolic pressure (PASP) was determined through the tricuspid regurgitation velocity gradient and inferior vena cava size and reactivity. LV ejection fraction (LVEF) (determined by biplane Simpson’s method) and global longitudinal strain (LVSlong, averaged strain measured in apical longitudinal, 4-chamber and 2-chamber view by 2D STE) were used as LV systolic parameters. LV diastolic parameter measurements included the ratio of mitral inflow early (E) and late (A) diastolic velocity (E/A), septal mitral annular early (E’) and late (A’) diastolic velocity, and E/E’.

### LA phasic strain by 2D STE

**Fig. 1:**
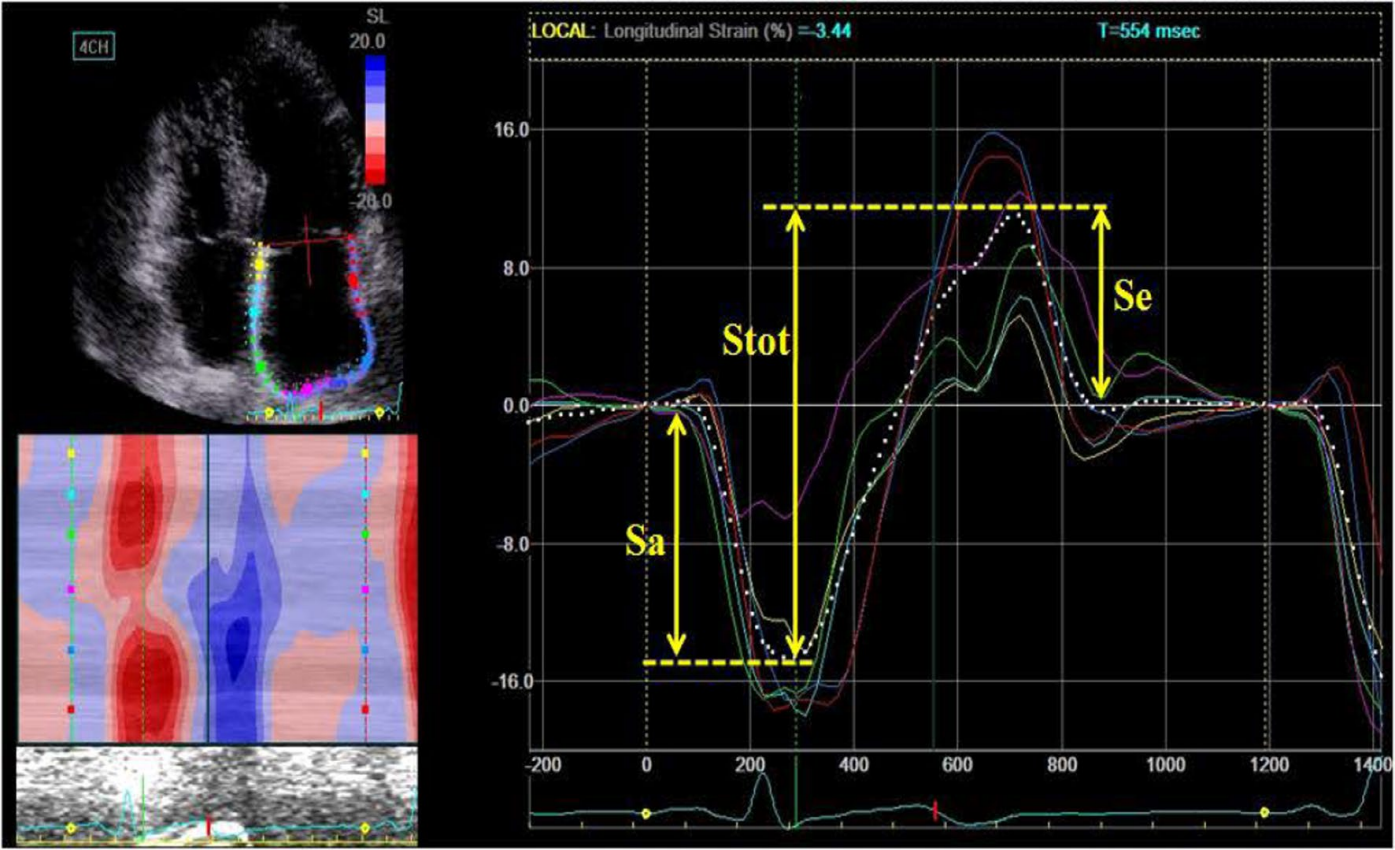
Left atrial phasic strain measurements from four-chamber apical view by 2D STE. Sa, peak strain at atrial contraction phase; Se, peak strain at early diastole phase; Stot, total atrial strain (Stot = Sa + Se)

Global LA strain were analyzed in apical 4-chamber view 2D image using commercialized software. 2D image of a representative cardiac cycle was selected, and the LA endocardial surface was manually traced by a point-and-click approach. An epicardial surface tracing was automatically generated by the system, creating a region of interest (ROI), which was manually adjusted to cover the full thickness of the LA wall. The software divided the ROI into 6 segments and generated segmental as well as global longitudinal LA strain curves. The onset of the *P* wave of the superimposed ECG was used as the reference point, which enabled the recognition of absolute peak strain during atrial contraction (Sa), peak strain at early diastole (Se) and total atrial strain (Stot = Sa + Se) in strain curve (Fig. 1). Sa was corresponding to LA contractile, Stot to reservoir and Se to conduit function, respectively [23–25].

### Repeatability and reproducibility

15 randomly selected cases were re-measured by the same observer blinded to previous measurements with 1 week interval, and by a second observer blinded to the first one’s results to assess intra- and inter- observer variability.

### Statistical analysis

Data were analyzed with the use of the statistical packages R (The R foundation; http://www.r-project.org; version 3.4.3) and Empower (R) (www.empowerstates.com, X&Y solutions, inc. Boston, Massachusetts). Continuous variables were presented as mean ± standard deviation (SD) and categorical variables as percentage rate (%). Differences among more than three groups were compared using one-way ANOVA and Dunnett’s test. Student t test was used in comparison between two groups. A Chi-square test was used to compare categorical values between groups. Univariate correlation followed multiple linear regression analyses were performed to examine the association of select clinical variables and echocardiographic findings with LA phasic strain indexes.

Given the differences in the clinical characteristics among the four study groups, propensity-score matching [Normal LA size group (16–34 ml/m^2^) and Severe LAE group (> 48 ml/m^2^), Normal LA size group (16–34 ml/m^2^) and Abnormal LA size group (Mild, Moderate and Severe LAE) (> 35 ml/m^2^), respectively] was used to adjust age, HT and CHD. Matching was performed with the use of 1:2 and 1:1 matching protocol, with a caliper width equal to 0.01 of the standard deviation of the logit of the propensity score. In the matched groups, paired comparisons were performed with the use of McNemar’s test for binary variables and a paired Student’s t-test for continuous variables. Univariate linear regression was performed in the matched groups.

All *P* values are two-sided and were considered statistically significant if less than 0.05.

## Results

### Demographic and echocardiographic characteristics of the high risk population according to left atrial volume index

A total of 770 subjects at cardiovascular high risk in the community underwent comprehensive transthoracic echocardiogram. 55 were excluded the current study including 22 with poor image quality (unable to obtain adequate tracking quality in more than two LA segments during offline strain analysis), 14 in atrial fibrillation at the time of echocardiography examination, and 19 with moderate or more mitral regurgitation or any degree of mitral stenosis. Among the 4314 segments analyzed in the remaining 715 subjects, the software was able to correctly track 4055 (94%) segments.

In this cross-sectional study, the average age was 66.56 ± 8.86 years and 354 (49.51%) of the subjects were male. The existed CVD and risk factors included CHD (9.09%), stroke (20.98%), peripheral vascular disease (8.67%), HT (80.28%), DM (30.21%), smoke (33.71%), obesity (20.42%) and dyslipidemia (49.51%). According to the abnormality thresholds and severity cutoffs of LAVI in ASE/EACVI recommendation in 2015 [19], subjects were grouped into normal LA size (LAVI 16–34 ml/m^2^), mild LA enlargement (LAVI 35–41 ml/m^2^), moderate LA enlargement (LAVI 42–48 ml/m^2^) and severe LA enlargement (LAVI > 48 ml/m^2^) group. Tables 1 and 2 present demographic and conventional echocardiographic characteristics of the four study groups. Among-group differences were found in age, heart rate, the prevalence of HT (most prevalent in mild LAE group), LVMI, LVEDD, diastolic indexes (E, E/A, E/E’, A’), LAEF and PASP. There were no differences in other risk factors and medication among groups.

**Table 1:** Demographic characteristics of the four study groups

Variable	Normal LA size (16–34 ml/m^2^, n = 507)	Mild LAE (35–41 ml/m^2^, n = 134)	Moderate LAE (42–48 ml/m^2^, n = 51)	Severe LAE (> 48 ml/m^2^, n = 23)	*P*
Age (years)	65.96 ± 9.11	67.75 ± 8.39	67.55 ± 7.16	70.65 ± 7.90	0.016
Gender, male	47.73%	48.51%	62.75%	65.22%	0.088
BSA (m^2^)	1.84 ± 0.16	1.82 ± 0.16	1.87 ± 0.18	1.85 ± 0.14	0.357
BMI (kg/m^2^)	25.56 ± 3.29	25.50 ± 3.27	25.57 ± 3.72	25.32 ± 3.12	0.986
SBP (mmHg)*	171.41 ± 19.06	177.13 ± 18.50	172.13 ± 21.24	184.74 ± 18.37	0.002
DBP (mmHg)*	98.83 ± 17.31	97.72 ± 17.39	97.11 ± 22.04	100.79 ± 18.05	0.814
HR (beats/min)	71.07 ± 10.94	66.46 ± 10.58	65.67 ± 8.93	62.13 ± 8.74	< 0.001
CHD	7.50%	12.69%	13.73%	13.40%	0.144
Stroke	20.71%	17.91%	25.49%	34.78%	0.256
Peripheral vascular disease	8.09%	8.96%	11.76%	13.04%	0.705
HT	76.73%	90.30%	88.24%	82.61%	0.002
Diabetes mellitus	31.95%	25.37%	31.37%	17.39%	0.257
Smoking	33.53%	28.36%	43.14%	47.83%	0.122
Obesity	20.32%	20.90%	21.57%	17.39%	0.979
Dyslipidemia	49.11%	52.24%	52.94%	34.78%	0.446
Aspirin	51.48%	50.00%	35.29%	47.83%	0.179
ACEI	7.30%	5.97%	7.84%	4.35%	0.898
ARB	3.36%	4.48%	5.88%	13.04%	0.117
Calcium antagonists	39.25%	44.78%	33.33%	43.48%	0.484
βblockers	6.71%	9.02%	9.80%	17.39%	0.223
Statins	0.99%	0.00%	3.92%	4.55%	0.056
Diuretics	3.35%	0.75%	5.88%	4.35%	0.257

Data are expressed as mean ± SD or as percentage

*BSA* Body surface area, *BMI* Body mass index, *SBP* Systolic blood pressure, *DBP* Diastolic blood pressure, *HR* Heart rate, *CHD* Coronary heart disease, *HT* Hypertension, *ACEI* Angiotensin-Converting Enzyme Inhibitor, *ARB* Angiotensin receptor blocker

*SBP, DBP were only recorded in HT participants. SBP, DBP value above are mean ± SD in HT participants

**Table 2:** Conventional echocardiographic characteristics of the four study groups

Variable	Normal LA size (16–34 ml/m^2^, n = 507)	Mild LAE (35–41 ml/m^2^, n = 134)	Moderate LAE (42–48 ml/m^2^, n = 51)	Severe LAE (> 48 ml/m^2^, n = 23)	*P*
LVEDD (cm)	4.59 ± 0.49	4.77 ± 0.49	4.88 ± 0.59	5.03 ± 0.73	< 0.001
LVEF (%)	63.77 ± 6.46	63.93 ± 6.98	62.41 ± 9.37	61.96 ± 11.01	0.345
LVSlong (%)	18.38 ± 3.13	18.63 ± 3.34	18.45 ± 4.63	17.37 ± 4.69	0.586
LVMI (g/m^2^)	83.34 ± 20.06	93.95 ± 23.82	94.36 ± 29.29	122.47 ± 38.41	< 0.001
E-wave (cm/s)	76.05 ± 18.72	80.57 ± 20.60	83.43 ± 20.29	91.13 ± 31.30	< 0.001
A-wave (cm/s)	94.29 ± 19.21	97.85 ± 29.33	91.84 ± 17.64	99.73 ± 20.38	0.167
E/A retio	0.83 ± 0.24	0.87 ± 0.31	0.93 ± 0.27	0.88 ± 0.20	0.033
E’ (cm/s)	6.08 ± 1.89	5.86 ± 1.73	5.98 ± 1.69	5.00 ± 1.02	0.040
A’ (cm/s)	10.43 ± 1.85	9.75 ± 1.85	9.58 ± 2.15	8.81 ± 1.94	< 0.001
E/e’	13.30 ± 4.10	14.72 ± 5.18	14.89 ± 5.57	18.74 ± 6.36	< 0.001
PASP (mmHg)	28.71 ± 6.33	31.08 ± 6.95	32.89 ± 9.02	33.25 ± 6.67	< 0.001
LAEF	0.54 ± 0.08	0.54 ± 0.08	0.50 ± 0.08	0.44 ± 0.13	< 0.001

Data are expressed as mean ± SD

*LVEDD* Left ventricular end-diastolic dimension, LVEF Left ventricular ejection fraction, *LVSlong* left ventricle global longitudinal strain, *LVMI* Left ventricular mass index, *PASP* Pulmonary artery systolic pressure, *LAEF* left atrial ejection fraction

### LA phasic strain in different LA size group

The comparison of LA strain among the study groups is shown in Table 3. From normal size, mild, moderate to severe LAE, Reservoir (Stot) is 21.74 ± 5.97, 20.75 ± 4.99, 20.49 ± 5.27, 17.75 ± 4.71, respectively (*P* = 0.003), and contractile (Sa) is 11.84 ± 3.92, 11.00 ± 3.29, 10.11 ± 2.57, 8.55 ± 2.88, respectively (*P* < 0.001). Reservoir (Stot) of Severe LAE group was significantly lower than that of Normal LA size group (*P* = 0.002). While contractile (Sa) indexes of the three groups were all significantly lower than that of Normal LA size group (*P* = 0.024, *P* = 0.002, *P* < 0.001, respectively) (Seen in Table 3). The conduit (Se) indexes are similar among the four different LA size groups.

**Table 3:** Left atrial global phasic strain in different LA size group

Variable	Normal LA size (16–34 ml/m^2^, n = 507)	Mild LAE (35–41 ml/m^2^, n = 134)	Moderate LAE (42–48 ml/m^2^, n = 51)	Severe LAE (> 48 ml/m^2^, n = 23)	*P*
Stot	21.74 ± 5.97	20.75 ± 4.99	20.49 ± 5.27	17.75 ± 4.71**	0.003
Se	9.90 ± 4.58	9.74 ± 3.81	10.38 ± 4.25	9.20 ± 3.06	0.719
Sa	11.84 ± 3.92	11.00 ± 3.29*	10.11 ± 2.57**	8.55 ± 2.88**	< 0.001

Data are expressed as mean ± SD or as percentage

*P < 0.05 versus Normal LA size group

**P < 0.01 versus Normal LA size group

### LA phasic strain between HT and non-HT groups with normal LA size

Within normal LA size group, subjects were further divided into HT and non-HT subgroups. The HT group had higher prevalence of stroke (23.91% vs. 10.17%, *p* < 0.001) and obesity (23.65% vs. 9.32%, *P* < 0.001) comparing with non-HT group. Differences between HT and non-HT group were also found in age (66.56 ± 8.82 vs. 64.00 ± 9.78 years, *P* = 0.007), body mass index (25.91 ± 3.30 vs. 24.44 ± 3.00 kg/ m^2^, *P* < 0.001), LVMI (84.94 ± 20.10 vs. 78.11 ± 19.10 g/m^2^, *P* = 0.001), A velocity (96.85 ± 18.35 vs. 85.81 ± 19.65 cm/s, *P* < 0.001), E’ (5.76 ± 1.63 vs. 7.12 ± 2.29 cm/s, *P* < 0.001), E/A (0.80 ± 0.21 vs. 0.94 ± 0.31, *P* < 0.001) and E/e’ (13.80 ± 4.10 vs. 11.67 ± 3.67, *P* < 0.001). There were no differences in other clinical and echocardiographic parameters. The comparison of clinical data and LA strain was shown in Table 4. Reservoir (Stot) and conduit (Se) indexes were worse in HT than non-HT subgroup, while contractile (Sa) index had no difference.

**Table 4:** Left atrial global phasic strain in HT and non-HT subgroups with normal left atrial size (n = 507)

Variable	Non-HT (n = 118)	HT (n = 389)	*P*
Age (year)	64.00 ± 9.78	66.56 ± 8.82	0.007
Male (%)	50.85%	46.79%	0.439
LAVI	25.15 ± 4.55	27.14 ± 4.60	< 0.001
LAEF	0.55 ± 0.07	0.54 ± 0.09	0.084
Stot	23.01 ± 6.02	21.35 ± 5.91	0.008
Se	11.17 ± 4.89	9.51 ± 4.41	< 0.001
Sa	11.84 ± 3.83	11.84 ± 3.95	0.993

Data are expressed as mean ± SD or as percentage

*HT* Hypertension, *LAVI* Left atrial volume index, *LAEF* left atrial ejection fraction

### Clinical and echocardiographic correlates of LA phasic strain and LAVI: regression analysis

Univariate correlations of LA strain includes demographic parameters (age, gender, body mass index, body surface area, heart rate, SBP and DBP), CVD (myocardial infarction, stroke, peripheral arterial disease), risk factors (HT, HT duration, grade of HT, DM, smoke, obesity and hyperlipidemia) and echocardiographic variables (LAVI, LVEDD, LVMI, LVEF and LVSlong, E/A, E/e’, E’, A’, LAEF and PASP). Stepwise multiple regression analysis showed that the independent determinants were: for Stot—age, DM, smoking, dyslipidemia, LVSlong and A’; for Se—HR, dyslipidemia, LVSlong and E’; and for Sa—age, gender, smoking, LAVI, LVSlong, E/A, E’ and A’ (Table 5).

**Table 5:** Associations of left atrial functional and structural characteristics

	Variables					
	Stot		Se		Sa	
	β	*P*	β	*P*	β	*P*
Age	− 0.064	0.021			− 0.040	0.031
Male						
HR	− 0.051	0.021	− 0.048	0.006		
DM	0.986	0.039			0.719	0.022
Smoking					− 1.183	0.001
Dyslipidemia						
LAVI	− 0.062	0.026			− 0.068	< 0.001
LVSlong	0.376	< 0.001	0.208	< 0.001	0.169	< 0.001
E/A					− 1.647	0.019
E’			0.637	< 0.001	− 0.441	< 0.001
A’	0.386	< 0.001			0.406	< 0.001
LAEF	12.316	< 0.001	7.596	< 0.001	4.709	0.012
Adjusted R^2^	0.196		0.204		0.178	

*HR* Heart rate, *DM* Diabetes mellitus, *LVSlong* Left ventricle global longitudinal strain, *LAEF* left atrial ejection fraction

Given that Stot and Se were both significantly decreased in HT subgroup with normal LA size, multiple regression analysis was performed. Stot showed a significant association with smoking, LVSlong, A’ and LAEF. While Se was associated with HR, LVSlong, E’ and LAEF (Additional file 1: Table S1).

Detailed descriptions of propensity-score matching were presented in Additional file 1: Figs. S1, S2, and Tables S2, S4. Larger LAVI was associated with lower Sa even after matching for age, HT and CHD (Additional file 1: Tables S3, S5), which was in accordance with the results in Table 5.

### Repeatability and reproducibility

The intra-observer intraclass correlation coefficient (ICC) and absolute difference for 2D STE measurements were Stot 0.97(1.62 ± 1.45%), Se 0.98(1.43 ± 0.76%) and Sa 0.92(1.17 ± 0.99%). And inter observer ICC and absolute difference were Stot 0.93 (2.07 ± 1.73%), Se 0.89 (2.50 ± 2.35%) and Sa 0.88 (1.76 ± 1.23%), respectively.

## Discussion

This cross-sectional study was aimed in exploring the relationship between LA phasic function (phasic strain measured by 2D STE) and LA size in a high cardiovascular risk community population, which was of clinical value for understanding LA performance and pathophysiology. Results showed that LA contractile index (Sa) deteriorated with LAE, LA reservoir index (Stot) deteriorated particularly in Severe LAE subjects, while conduit index (Se) roughly unchanged during LAE, suggesting that LAE was accompanied with redistributed phasic function. Moreover, compared with non-HT subjects, LA conduit (Se) and reservoir (Stot) indexes appeared to be impaired in HT participants despite maintaining normal LA size, which maybe a compensatory response often accompanied by impaired LV relaxation.

LA is far from being a simple passive transport chamber. LA functions as a reservoir during LV systole and isovolumic relaxation, receiving blood from the pulmonary veins and storing energy in the form of pressure. This atrial function is modulated by LV contraction, through the descent of the LV base during systole, by right ventricular systolic pressure transmitted through the pulmonary circulation, and by LA properties (ie, relaxation and chamber stiffness) [26]. LAVI is the only LA parameter evaluated in general clinical practice to reflect LA function. However, LA strain can also be used as a surrogate measurement of LA function [27] and prior studies showed that it might be a predictor of adverse outcomes among HT patients [3, 28] or other CVD high risk patients [11, 13, 29, 30]. A 7.9 year follow-up study [31] showed that LA reservoir and conduit strains were impaired in the stroke group compared with non-stroke group with similar LAV. Another longitudinal study [32] also provided support for LA strain’s prediction value to new-onset AF in heart failure patients. In a community-based general population study, LA reservoir strain below 23% was proved to be an independent predictor of cardiac events including AF, acute coronary syndrome, heart failure [9]. Daniel et al. reported that abnormal LA strain was observed in patients of left ventricular diastolic dysfunction with normal LAVI [10]. Moreover, worse LA strain was significantly related to worse New York Heart Association functional class, even in the participants with normal LAVI [10]. These recent studies demonstrate that LA strain (LA phasic function) might be of high clinical significance, relatively sensitive and superior to LAVI in measuring and detecting unfavorable outcomes.

**Fig. 2:**
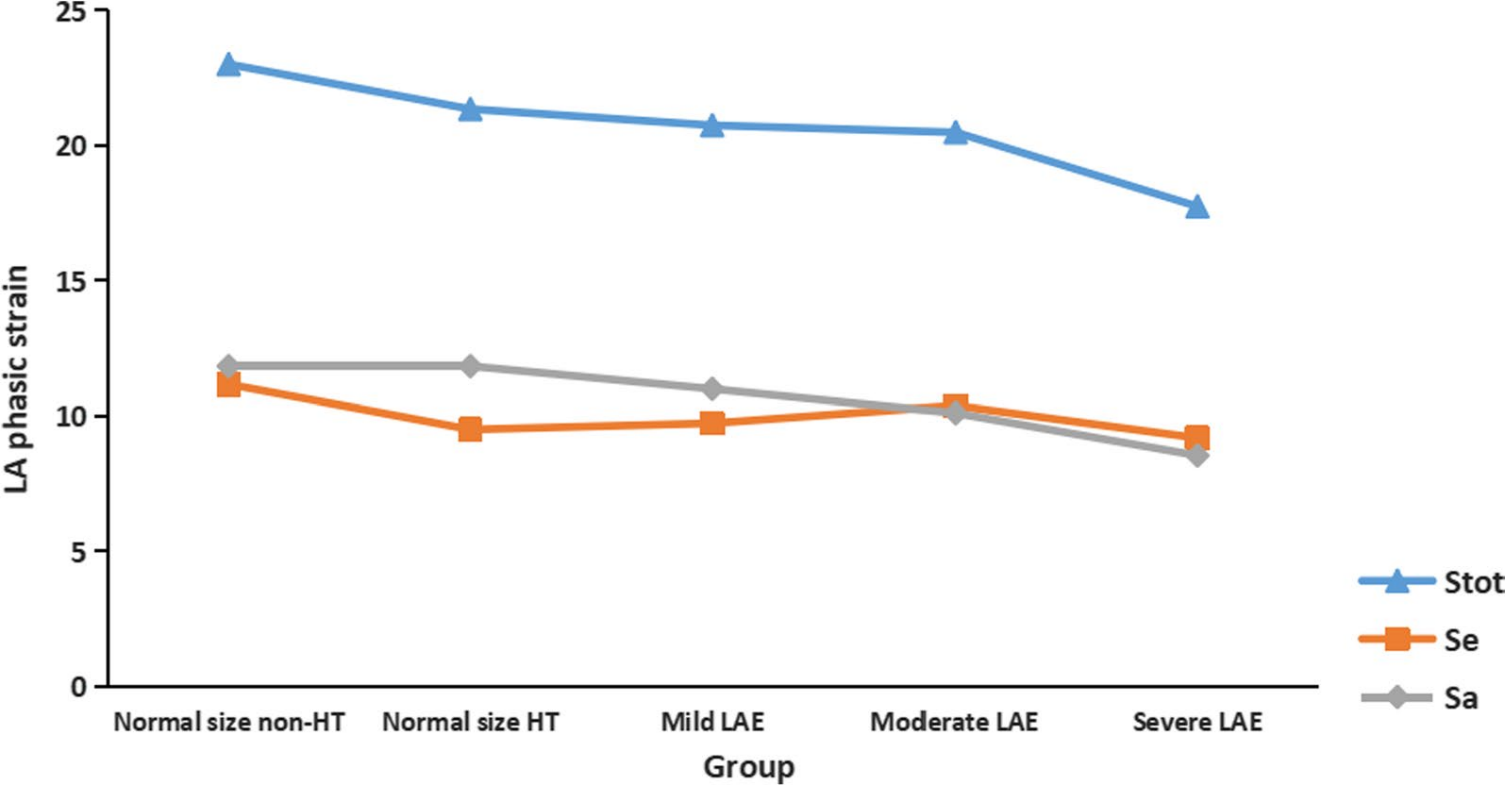
LA phasic strain in study groups

Figure 2 draws the spectrum of the LA structural and functional remodeling from normal size to severe LAE by phasic strain. The reservoir function (Stot) was impaired under stressors (i.e. HT in this population) even before LAE, which may indicate that not only wall stretching (which meet the Frank-Starling law, e.g., LA Strain impaired in advanced stretching) but also potential histological change (e. g., fibrosis in HT) can be reflected by LA strain. The conduit function (Se) is damaged in HT subjects before LAE, and remains unchanged during gradually LA enlargement, which suggests Se correlates more with pathophysiological condition rather than LA size. The possible reason is that the conduit is a passive press relying on LV relaxation and relatively not affected by LA structural remodeling, so in this population we observed no difference of Se among normal size and LAE groups, while in subgroup analysis it reduced in HT subjects, which commonly impairs LV relaxation. Moreover, many previous researches focused on LA function in HT patients. Miyoshi et al. found LA reservoir and conduit function reduced in HT patients than those in control, but LA contractile function was similar in the two groups [33]. However, another Chinese cross-sectional study reported that these three indexes of LA function all impaired in HT group [34]. Disparities among these reports may result from different grade of HT, age, duration of HT, grade of HT, medication and the control of blood pressure [35, 36].

In short, LA strain (Stot, Se, Sa) are relatively well-characterized surrogate marker of atrial function which play an essential role in early detection of subclinical LA dysfunction [13, 37–40]. This cross-sectional study provides incremental evidence over prior papers by the direct analysis of the association between LA size and function, as well as by the evaluation of the order of presence of LA dysfunction and LAE in HT patients. At present, despite more and more studies focus on these echo parameters, risk stratification and clinical strategies do not exploit them into clinical practice. Evidences concerning the spectrum of LA phasic function provide new insights and useful adjunctive information into the recognition of LA function and structure, especially in HT patients. LA strain (LA phasic function) might be promising predictors in the future.

## Limitation

The off line 2D STE software used in this study was not verified by other standard method in LA phasic function evaluation. LAV were not compared with three-dimensional echocardiographic or cardiac MRI measurements. We used only apical 4 chamber view in LA function evaluation instead averaging 2 or 3 apical views data as other researchers did, while the data should be comparable among groups in deducing our results. And we used the image database stored in standard views, while the LA and LV may not coaxial in the long axis, so the precise designed study in LA analysis should collect images focusing on LA. The clinical and prospective significance of LA phasic function remodeling needs to be confirmed in future studies.

## Conclusions

In this high cardiovascular risk community population study, we find that LA phasic function remodeling occurs before LA enlargement and continues with LAE. LA reservoir function is impaired under stressors (i.e. HT in this population) before LAE, particularly in Severe LAE subjects. The conduit function is damaged (in HT subjects) before LAE, while remains unchanged during gradually LAE. The LA contractile function deteriorates with LAE.

## Supplementary Information

**Additional file 1: Table S1.** Associations of left atrial functional and structural characteristics in HT subgroup with normal LA size (n = 389). **Fig. S1.** 1:2 matching according to age, HT and CHD between Normal LA size group (16-34 ml/ m^2^) and Severe LAE group (> 48ml/ m^2^) (caliper width = 0.05). **Table S2.** Characteristics before and after Propensity Score-Matching according to age, HT and CHD between Normal LA size group and Severe LAE group. **Table S3.** Association of LA size with Strain in the Propensity Score-Matching Group (Normal LA size group and Severe LAE group). **Fig. S2.** 1:1 matching according to age, HT and CHD between Normal LA size group (16-34 ml/ m^2^) and Abnormal LA size group (Mild, Moderate and Severe LAE ) (> 35ml/ m^2^) (caliper width = 0.05). **Table S4.** Characteristics before and after Propensity Score-Matching according to age, HT and CHD between Normal LA size group and Abnormal LA size group. **Table S5.** Association of LA size with Strain in the Propensity Score-Matched Group (Normal LA size group and Abnormal LA size group).

## Data Availability

The data and materials used in this study are available from the corresponding author on reasonable request.

## Abbreviations

LA: Left atrial
LAE: LA enlargement
2D STE: Two-dimensional speckle-tracking echocardiography
HT: Hypertension
LV: Left ventricular
CVD: Cardiovascular diseases
BMI: Body mass index
DM: Diabetes mellitus
BSA: Body surface area
LAVI: LA volume index
PASP: Pulmonary artery systolic pressure
LVEF: LV ejection fraction
ROI: Region of interest
SD: Standard deviation
ICC: Intraclass correlation coefficient
LAEF: Left atrial ejection fraction
