# Supplementary material for: Left atrial phasic function remodeling during its enlargement: a two-dimensional speckle-tracking echocardiography study
Source: BMC Cardiovasc Disord. 2022 May 19;22:231. doi: 10.1186/s12872-022-02672-z (PMC9118856; doi:10.1186/s12872-022-02672-z)
Supplement: Supplementary file 1 — Additional file 1: Table S1. Associations of left atrial functional and structural characteristics in HT subgroup with normal LA size (n = 389). Fig. S1. 1:2 matching according to age, HT and CHD between Normal LA size group (16-34 ml/ m2) and Severe LAE group (> 48ml/ m2) (caliper width = 0.05). Table S2. Characteristics before and after Propensity Score-Matching according to age, HT and CHD between Normal LA size group and Severe LAE group. Table S3. Association of LA size with Strain in the Propensity Score-Matching Group (Normal LA size group and Severe LAE group). Fig. S2. 1:1 matching according to age, HT and CHD between Normal LA size group (16-34 ml/ m2) and Abnormal LA size group (Mild, Moderate and Severe LAE ) (> 35ml/ m2) (caliper width = 0.05). Table S4. Characteristics before and after Propensity Score-Matching according to age, HT and CHD between Normal LA size group and Abnormal LA size group. Table S5. Association of LA size with Strain in the Propensity Score-Matched Group (Normal LA size group and Abnormal LA size group). [file 12872_2022_2672_MOESM1_ESM.docx]

**Additional File**

Title : Left atrial phasic function remodeling during its enlargement: A two-dimensional speckle-tracking echocardiography study

Chuyun Chen, Ying Yang*, Wei Ma, Litong Qi, Baowei Zhang, Yan Zhang

- **Table S1.** Associations of left atrial functional and structural characteristics in HT subgroup with normal LA size (n=389).
- **Figure S1.** 1:2 matching according to age, HT and CHD between Normal LA size group (16-34ml/ m^2^) and Severe LAE group (>48ml/ m^2^) (caliper width = 0.05).
- **Table S2.** Characteristics before and after Propensity Score-Matching according to age, HT and CHD between Normal LA size group and Severe LAE group.
- **Table S3.** Association of LA size with Strain in the Propensity Score-Matching Group (Normal LA size group and Severe LAE group).
- **Figure S2.** 1:1 matching according to age, HT and CHD between Normal LA size group (16-34ml/ m^2^) and Abnormal LA size group (Mild, Moderate and Severe LAE ) (>35ml/ m^2^) (caliper width = 0.05).
- **Table S4.** Characteristics before and after Propensity Score-Matching according to age, HT and CHD between Normal LA size group and Abnormal LA size group.
- **Table S5:** Association of LA size with Strain in the Propensity Score-Matched Group (Normal LA size group and Abnormal LA size group).

**Table S1:** Associations of left atrial functional and structural characteristics in HT subgroup with normal LA size (n=389).

| Variables | | | | |
| --- | --- | --- | --- | --- |
|  | Stot | | Se | |
|  | β | p | β | p |
| Age |  |  |  |  |
| Male |  |  |  |  |
| HR |  |  | -0.052 | 0.031 |
| DM |  |  |  |  |
| Smoking | -2.299 | 0.009 |  |  |
| Dyslipidemia |  |  |  |  |
| LAVI |  |  |  |  |
| LVSlong | 0.414 | <0.001 | 0.269 | 0.001 |
| E/A |  |  |  |  |
| E’ |  |  | 0.708 | <0.001 |
| A’ | 0.366 | 0.048 |  |  |
| LAEF | 12.513 | 0.003 | 6.919 | 0.022 |
| Adjusted R^2^ | 0.147 | | 0.193 | |

***HR*** Heart rate, ***DM*** Diabetes mellitus, ***LVSlong*** Left ventricle global longitudinal strain, ***LA*** left atrial ejection fraction

**Figure S1:** 1:2 matching according to age, HT and CHD between Normal LA size group (16-34ml/m^2^) and Severe LAE group (>48ml/m^2^) (caliper width = 0.01).


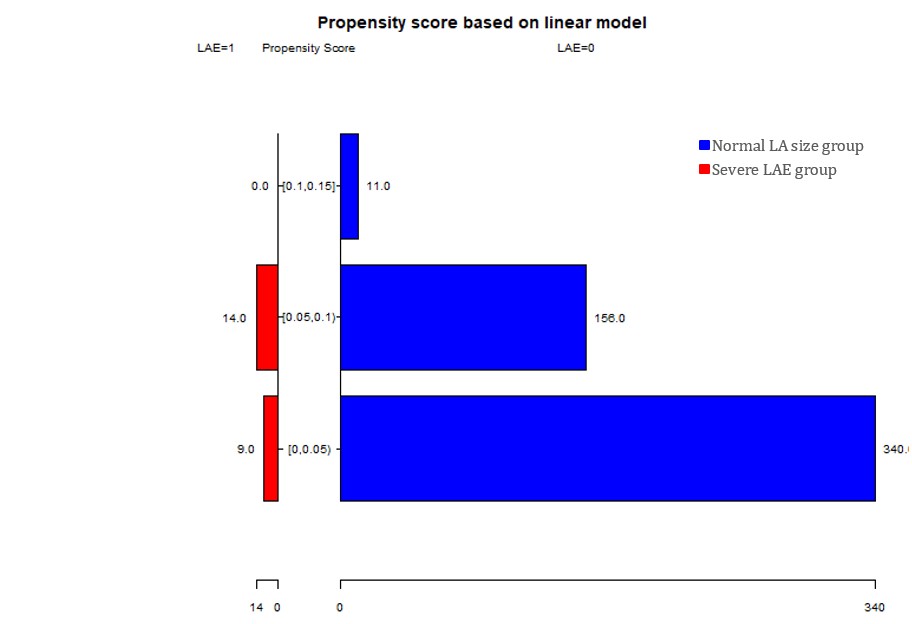


***LAE =1*** Severe LAE group (>48ml/m^2^), ***LAE =0*** Normal LA size group (16-34ml/m^2^).

**Table S2:** Characteristics before and after Propensity Score-Matching according to age, HT and CHD between Normal LA size group and Severe LAE group.

|  | **Before Matching** | | | | **After Matching** | | |  |  |
| --- | --- | --- | --- | --- | --- | --- | --- | --- | --- |
|  | | Normal LA size  (16-34ml/m^2^)  (n=507) | Severe LAE  (>48ml/m^2^)  (n=23) | Standized Difference | Normal LA size  (16-34ml/m^2^)  (n=46) | | Severe LAE  (>48ml/m^2^)  (n=23) | Standized Difference | |
| Age (year) | | 65.96±9.11 | 70.65±7.90 | 0.55 | 67.15±9.57 | | 70.65±7.90 | 0.40 | |
| HT | | 76.73% | 82.61% | 0.15 | 69.60% | | 82.60% | 0.31 | |
| CHD | | 7.50% | 13.40% | 0.18 | 13.00% | | 13.00% | 0.00 | |
| Stot | | 21.74±5.97 | 17.75±4.71 | 0.74 | 22.16±6.39 | | 17.75±4.71 | 0.79 | |
| Se | | 9.90±4.58 | 9.20±3.06 | 0.18 | 9.69±4.35 | | 9.20±3.06 | 0.13 | |
| Sa | | 11.84±3.92 | 8.55±2.88 | 0.96 | 12.47±3.90 | | 8.55±2.88 | 1.14 | |

***HT*** Hypertension, ***CHD*** Coronary Heart Disease.

**Table S3:** Association of LA size with Strain in the Propensity Score-Matching Group (Normal LA size group and Severe LAE group).

| Group | Strain | **Before Matching** | | | **After Matching** | |
| --- | --- | --- | --- | --- | --- | --- |
|  |  | β (95%CI) | *P* | β (95%CI) | | *P* |
| Normal LA size (16-34ml/m^2^) |  | ref | | ref | | |
| Severe LAE (>48ml/m^2^) | Stot | -4.41 (-6.44, -2.38) | <0.001 | -3.92 (-6.03, -1.81) | | <0.001 |
|  | Se | -0.49 (-2.06, 1.08) | 0.543 | 0.13 (-1.35, 1.60) | | 0.867 |
|  | Sa | -3.92 (-5.14, -2.71) | <0.001 | -4.05 (-5.38, -2.72) | | <0.001 |

Propensity score matching for age, hypertension(HT), and coronary heart disease (CHD).

**Figure S2:** 1:1 matching according to age, HT and CHD between Normal LA size group (16-34ml/m^2^) and Abnormal LA size group (Mild, Moderate and Severe LAE ) (>35ml/m^2^) (caliper width = 0.01).

**
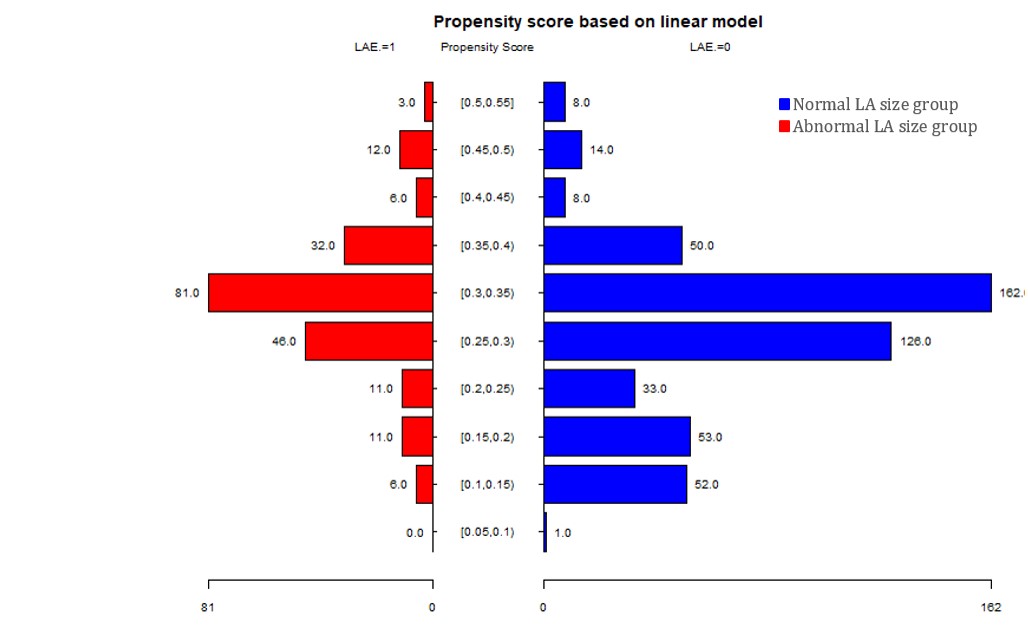
**

***LAE. =1*** Abnormal LA size group (>35ml/m^2^), ***LAE. =0*** Normal LA size group (16-34ml/m^2^).

**Table S4:** Characteristics before and after Propensity Score-Matching according to age, HT and CHD between Normal LA size group and Abnormal LA size group.

|  | **Before Matching** | | | | **After Matching** | | |
| --- | --- | --- | --- | --- | --- | --- | --- |
|  | Normal LA size  (16-34ml/m2)  (n=507) | Abnormal LA size  (>35ml/m2) (n=208) | Standized Difference | Normal LA size  (16-34ml/m2)  (n=207) | | Abnormal LA size  (>35ml/m2) (n=207) | Standized Difference |
| Age (year) | 65.96±9.11 | 68.02±8.07 | 0.24 | 67.13±8.41 | | 68.02±8.09 | 0.11 |
| HT | 76.73% | 88.94% | 0.33 | 86.00% | | 88.90% | 0.09 |
| CHD | 7.50% | 12.98% | 0.18 | 14.00% | | 12.60% | 0.04 |
| Stot | 21.74±5.97 | 20.35±5.09 | 0.25 | 20.84±5.88 | | 20.36±5.10 | 0.09 |
| Se | 9.90±4.58 | 9.84±3.85 | 0.01 | 9.17±4.22 | | 9.84±3.86 | 0.17 |
| Sa | 11.84±3.92 | 10.51±3.17 | 0.37 | 11.67±3.91 | | 10.52±3.18 | 0.32 |

***HT*** Hypertension, ***CHD*** Coronary Heart Disease.

**Table S5:** Association of LA size with Strain in the Propensity Score-Matched Group (Normal LA size group and Abnormal LA size group).

| Group | Strain | **Before Matching** | | | **After Matching** | |
| --- | --- | --- | --- | --- | --- | --- |
|  |  | β (95%CI) | *P* | β (95%CI) | | *P* |
| Normal LA size (16-34ml/m2) |  | ref | | ref | | |
| Abnormal LA size (>35ml/m^2^) | Stot | -0.48 (-1.51, 0.56) | 0.367 | -0.37 (-1.40, 0.67) | | 0.486 |
|  | Se | 0.67 (-0.07, 1.42) | 0.076 | 0.75 (0.01, 1.50) | | 0.048 |
|  | Sa | -1.15 (-1.83, -0.48) | <0.001 | -1.12 (-1.79, -0.45) | | 0.001 |

Propensity score matching for age, hypertension(HT), and coronary heart disease (CHD).
